# Constructing an Efficient *Bacillus subtilis* Spore Display by Using Cohesin−Dockerin Interactions

**DOI:** 10.3390/molecules26041186

**Published:** 2021-02-23

**Authors:** He Wang, Xiaomin Jiang, Yongchang Qian, Lianghong Yin

**Affiliations:** 1 School of Grain Science and Technology, Jiangsu University of Science and Technology, Zhenjiang 212100, China; 2 School of Agricultural and Food Sciences, Zhejiang Agriculture and Forestry University, Hangzhou 311300, China; jiang_xiaomin@hs-biopharm.com; 3 School of Forestry and Biotechnology, Zhejiang Agriculture and Forestry University, Hangzhou 311300, China; qian3906@zafu.edu.cn (Y.Q.); ylh4@163.com (L.Y.)

**Keywords:** spore display, *Bacillus subtilis*, cohesin dockerin interaction, β-galactosidase, display efficiency

## Abstract

*Bacillus subtilis* spore display has become a field of increasing interest in the past two decades. To improve the efficiency of *B. subtilis* spore display, its directed modification was performed based on the cellulosome architecture by introducing onto them divergent cohesin (Coh) modules that can specifically bind to the target enzyme bearing the matching dockerins (Doc). In this study, five different pairs of cohesins and dockerins, selected from four cellulolytic microbes, were examined for their capabilities in displaying a tetrameric enzyme β-galactosidase from *Bacillus stearothermophilus* IAM11001 on the surface of *B. subtilis* WB600 spores. Immunofluorescence microscopy, western blotting, dot blotting, and enzyme assay was applied to confirm its surface expression. All the resultant five Coh–Doc based spore display can hydrolyze *o*-nitrophenyl-β-D-galactopyranoside. Further, the optimized Coh–Doc based spore display exhibited the highest display efficiency. Overall, the results of current study may open new perspectives on the use of Coh–Doc interaction, which will find application in improving the efficiency of *B. subtilis* spore display.

## 1. Introduction

It has been 20 years since Isticato and colleagues described the use of CotB to anchor the C-terminal fragment of tetanus toxin on the *Bacillus subtilis* spores for developing a vaccine against tetanus [1]. *B. subtilis* spore surface display, referring to the expression of desired enzyme or antigen (target protein) on the surface of spore via fusion with a spore coat protein (anchoring motif), has been proven to be a powerful tool in fields of vaccine development, enzyme immobilization, as well as the delivery of cancer drug and human-related proteins [2,3]. The *B. subtilis* spore surface display offers natural advantages that include high safety, outstanding stability, and unique ability to display a broad variety of biomolecules, like antigens, tetrameric enzymes and proteins [4].

Currently, spore surface display can be accomplished through non-genetic and genetic approaches [5]. The non-genetic method highly depends on passive adsorption between the spore surface and the target protein, which involves electrostatic forces, hydrogen bonding, and hydrophobic interactions. However, those nonspecific affinities lead to uncontrollable adsorption, and the storage stability of the adsorbed protein is sometimes unsatisfactory [6]. The genetic method is completed by directly fusing anchoring motifs such as CotB, CotC, and CotG with target proteins. In this manner, a strong covalent bond between the anchoring motif and target protein can be created. However, the loss of catalytic activity is challenging, since most of the spore displayed proteins are buried in the coat layer, which makes it inaccessible for the binding of substrates [7].

Despite the great strides achieved in the past two decades, *B. subtilis* spore display still suffers from low expression level of the protein of interest, thus rendering it unsuitable for industrial applications. Tavassoli et al. [8] observed that the CotC deficient spores displaying *B. subtilis* 168 β-galactosidase retained 1.6-fold higher residual activity than the wild-type spores after three reuses, although both exhibited the same initial enzyme activity and expression level. The work of Nguyen and Schumann [9] also indicated that the isopropyl-β-D-thiogalactoside (IPTG)-inducible promoter *P_Sgrac_* could greatly increase the expression of *Bacillus amyloliquefaciens* α-amylase Q (AmyQ), but it gave the lowest activity (1.5 U) as compared to those obtained with *P_grac_* (3 U) and *P_cotB_* (2.5 U). It is noted that there was a big difference in the number of CotB∆–AmyQ molecule displayed per spore between the *P_grac_* and *P_cotB_* promoter, in which the former was calculated to be almost two times higher than the latter. Collectively, these observations suggest that a small portion of fusion proteins could exist in an enzymatically inactive form surrounding the spore surface, a restriction likely be due to the result of low catalytic efficiency arising from the steric hindrance between anchoring motif and the displayed enzyme.

Cellulosomes are extracellular large multienzyme complexes that are commonly produced by many different anaerobic cellulolytic bacteria [10]. Each of them contains one or more cohesin modules that interact with complementary modules termed dockerins. The cohesin-dockerin (Coh–Doc) interaction is considered to be one of nature’s strongest bimolecular interactions. Furthermore, the binding affinity between cohesin and dockerin is not influenced by the fused enzyme and the linker [11]. The architecture and unique subunit arrangement of the cellulosome can have the genetic potential to create a robust *B. subtilis* spore display platform by the Coh–Doc interaction. Inspired from these observations, the first evidence is the expression of *Escherichia coli* β-galactosidase on the spore surface with a controllable manner through Coh–Doc interaction [7]. Introducing Coh–Doc interaction might relieve the spatial barrier between the anchoring motif and the displayed protein, thus contributing to its elevated catalytic efficiency.

**Figure 1:**
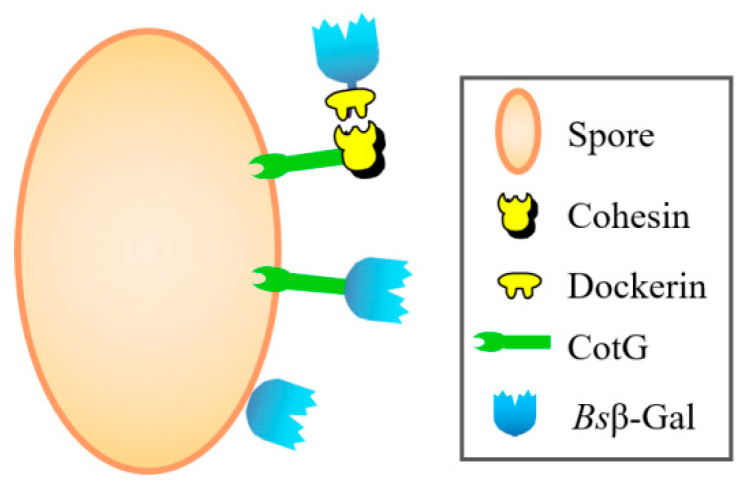
Scheme of three different types of *Bs*β–Gal *B. subtilis* spore display formats.

The objective of this research was to improve the performance of *B. subtilis* spore display by grafting an appropriated Coh–Doc system between anchoring motif and protein of interest. For this purpose, five different Coh–Doc modular pairs from different bacterial origins were utilized. A CotG-mediated *B. subtilis* spore display was engineered using β-galactosidase from *Bacillus stearothermophilus* IAM11001 (*Bs*β–Gal) as a model. The surface-expression of *Bs*β–Gal on the spores was demonstrated and its display efficiency was compared to conventional spore display based on direct fusion strategy and native protein display without anchoring motif (Figure 1).

## 2. Results

### 2.1. Generation of Coh–Doc Based Spore Surface Display Systems

To identify the most efficient Coh–Doc module for the display of *Bs*β–Gal on the surface of *B. subtilis* spores, CotG, which has been proved to facilitate *Bacillus* spore surface display of *Bs*β–Gal in our previous research [12], was employed as an anchoring motif. Meanwhile, three representative types of Coh–Doc modules, namely type I and type II Coh–Doc complexes from *Clostridium thermocellum* ATCC 27405, type I Coh–Doc complexes from *Clostridium cellulovorans* DSM 743B and *Clostridium cellulolyticum* ATCC 35319 and type III Coh–Doc complex from *Ruminococcus flavefaciens* FD-1, were selected. All the five selected cohesin genes were obtained by PCR and cloned to the C-terminus of CotG of pEB03-cotG, which was tagged with the FLAG sequence at the 3′-end of the cohesin gene. Following the transformation of wild type *B. subtilis* WB600 with the display plasmid, recombinant spores carrying CotG-fused cohesin were harvested. Besides, bgaB gene encoding a model protein *Bs*β–Gal was also PCR amplified and ligated into pET-28a, generating pET-28a-bgaB. Dockerin gene was amplified and fused to the C-terminal end of bgaB. In addition, the linker GGGGS was inserted between *Bs*β–Gal and its dockerin module. After transformation into *E. coli* BL21(DE3), different types of *Bs*β–Gal–Docs were purified by Ni-affinity chromatography column and confirmed by SDS-PAGE analysis. To examine whether the *Bs*β–Gal could be assembled onto the spore surface, each of the dockerin fused *Bs*β–Gal–Doc was incubated with spores surface-displaying a fusion of CotG and corresponding cohesin. After the same period of incubation, the assembly of *Bs*β–Gal onto the spore surface was achieved by Coh–Doc interaction.

### 2.2. Demonstration of Coh–Doc Based Spore Surface Display Using Immunofluorescence Analysis

**Figure 2:**
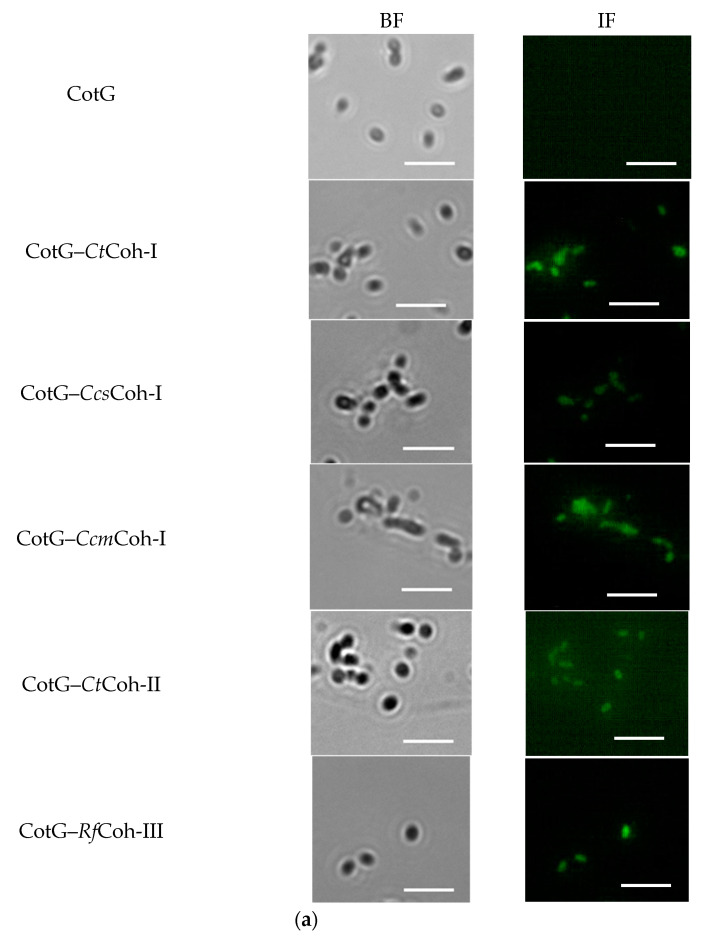

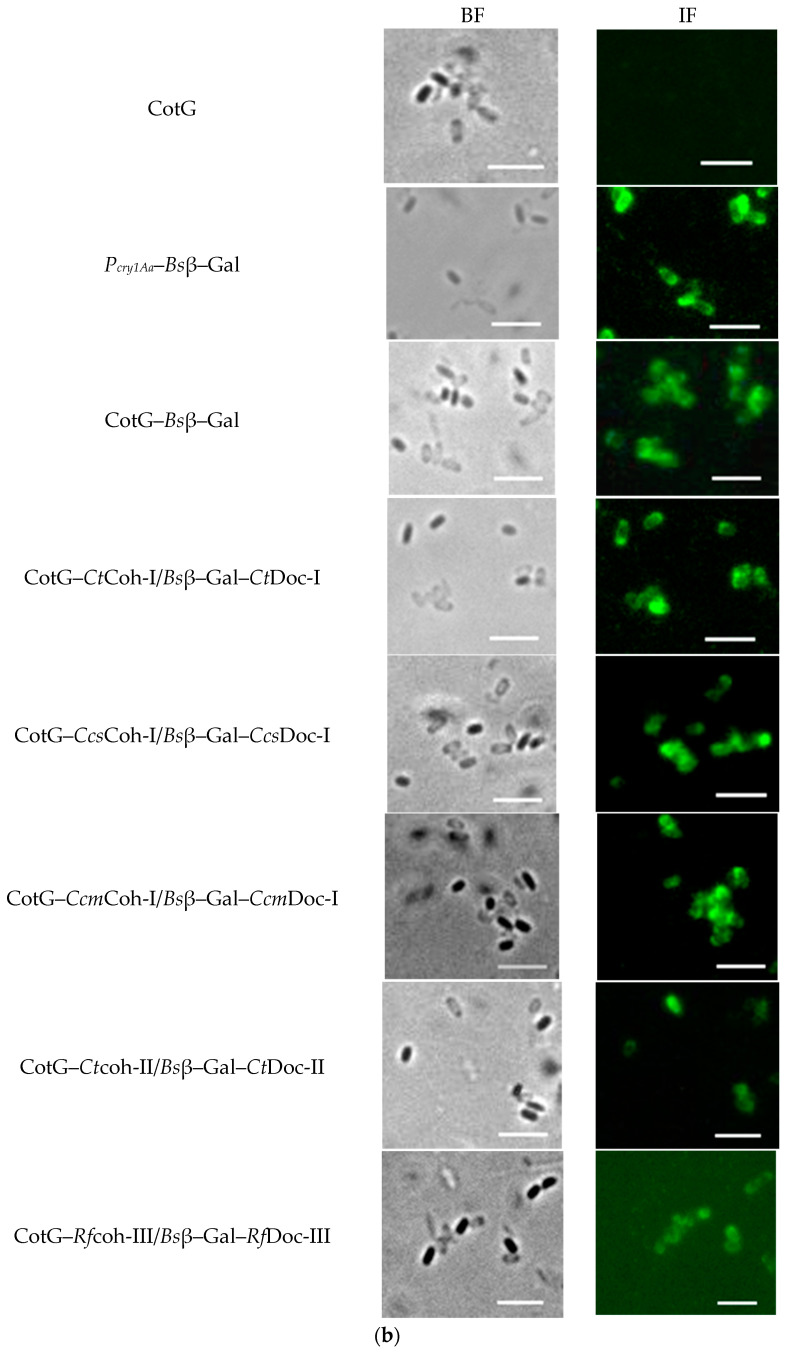
Identification of surface-expression of CotG–Coh (**a**) and CotG–Coh/*Bs*β–Gal–Doc (**b**) by immunofluorescence microscopy analysis. After being labeled with rabbit polyclonal anti–*Bs*β–Gal antibody, purified spores carrying CotG–Coh were imaged with fluorescent microscopy. The same procedure was applied on the spores carrying CotG–Coh/*Bs*β–Gal–Doc, except that they were labeled with anti–*Bs*β–Gal–Alexa Fluor 488. Spores obtained with *B. subtilis* WB600/pEB03-cotG served as a negative control. BF and IF, bright field and immunofluorescence images, respectively. Scale bar, 20 μm.

The purified spores of CotG–Coh were first labeled with Alexa Fluor 488-conjugated anti–FLAG antibody, and then observed using immunofluorescence microscopy. As shown in Figure 2a, no detectable signal was observed on *B. subtilis* WB600 containing pEB03-cotG, however, all five recombinant spores presented strong fluorescence signals, suggesting the successful expression of cohesin on the spore surface. To testify whether *Bs*β–Gal–Doc was anchored on the spore surface via interaction between cohesin and dockerin, the localization of CotG–Coh/*Bs*β–Gal–Doc was also assayed using immunofluorescence analysis. As a control, *B. subtilis* WB600 harboring pEB03-cotG alone showed no detectable fluorescence. By contrast, five Coh–Doc based spore displayed *Bs*β–Gals, together with CotG–*Bs*β–Gal and *P_cry1Aa_*–*Bs*β–Gal exhibited much stronger fluorescence signals than the control (Figure 2b), supporting that *Bs*β–Gal, recognized by the antibody, resided on the surface of spores.

### 2.3. Confirmation of Coh–Doc Based Spore Surface Display

**Figure 3:**
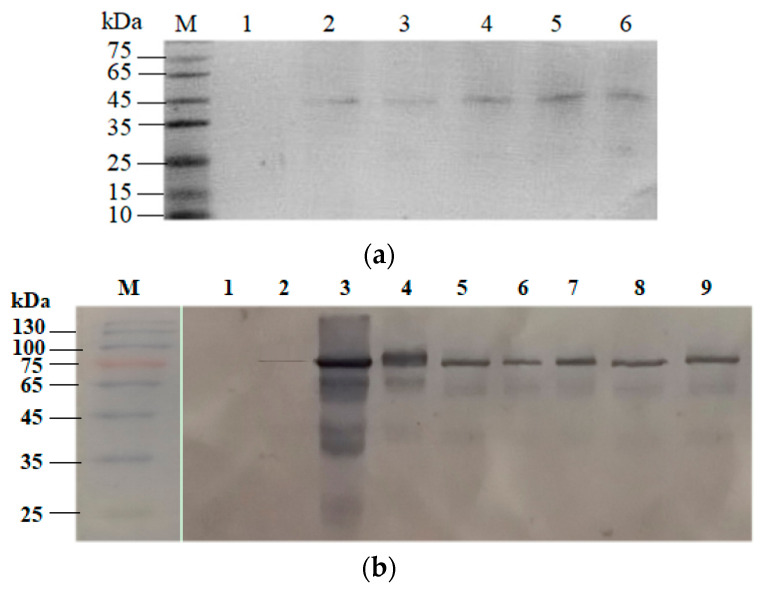
Western blotting analysis of immobilization of *Bs*β–Gal on the spores via Coh–Doc interaction. (**a**) Expression of CotG–Coh was analyzed using anti–FLAG as the primary antibody. M, standard protein marker; 1, CotG; 2, CotG–*Ct*Coh-I; 3, CotG–*Ct*Coh-II; 4, CotG–*Ccs*Coh-I; 5, CotG–*Ccm*Coh-I; 6, CotG–*Rf*Coh-III. (**b**) Expression of CotG–Coh/*Bs*β–Gal-Doc was detected using anti–*Bs*β–Gal–HRP. M, prestained standard protein marker; 1, CotG; 2, *Bs*β–Gal–*Ct*Doc-I (adsorbed onto the wild type spores); 3, *P_cry1Aa_*–*Bs*β–Gal; 4, CotG–*Bs*β–Gal; 5, CotG–*Ct*Coh-I/*Bs*β–Gal–*Ct*Doc-I; 6, CotG–*Ct*coh-II/*Bs*β–Gal–*Ct*Doc-II; 7, CotG–*Ccs*Coh-I/*Bs*β–Gal–*Ccs*Doc-I; 8, CotG–*Ccm*Coh-I/*Bs*β–Gal–*Ccm*Doc-I; 9, CotG–*Rf*coh-III/*Bs*β–Gal–*Rf*Doc-III.

CotG–Coh/*Bs*β–Gal–Doc was further characterized by the western blotting technique. Initial investigation into the surface expression of CotG–Coh was performed using anti–FLAG antibody. From the results shown in Figure 3a, a specific major band of approximately 45 kDa matching the theoretical molecular weight of CotG fused cohesin was detected in all five CotG–Coh samples, while *B. subtilis* WB600 harboring pEB03-cotG presented no similar protein band, suggesting that the specific band was target protein contained FLAG tag. The western blotting analysis verified successful expression of different CotG-fused cohesins on the spores.

Then, the presence of CotG–Coh/*Bs*β–Gal–Doc on the spores was examined through a western blotting experiment using goat anti-rabbit *Bs*β–Gal secondary antibody. The results showed single bands at the expected MWs for *P_cry1Aa_*–*Bs*β–Gal (97 kDa), CotG–*Bs*β–Gal (102 kDa), and *Bs*β–Gal–Doc (85~97 kDa). Figure 3b shows that CotG–Coh/*Bs*β–Gal–Doc was immobilized on the spores, since only one main characteristic band was found on the gel for all the seven different spores displayed *Bs*β–Gals. However, no similar protein bands were detected in the loading well for the wild type spores. Furthermore, the expression level of *Bs*β–Gal identified in all spore display systems were very higher than that of adsorbed treatment sample. From the above, the experimental data proofed the correct expression of *Bs*β–Gal based on five different Coh–Doc interactions on the surface of the spores. The five different types of Coh–Doc based spore displayed *Bs*β–Gals exhibited similar expression level, but lower than that of CotG–*Bs*β–Gal or *P_cry1Aa_*–*Bs*β–Gal, which was confirmed via the western blotting method. Among Coh–Doc based spore display systems, CotG–*Ccm*Coh-I/*Bs*β–Gal–*Ccm*Doc-I with the highest expression level and CotG–*Rf*Coh-III/*Bs*β–Gal–*Rf*Doc-III with the lowest expression level were chosen for further evaluation.

### 2.4. Effect of Coh–Doc Pair Type on Spore Display Efficiency

**Figure 4:**
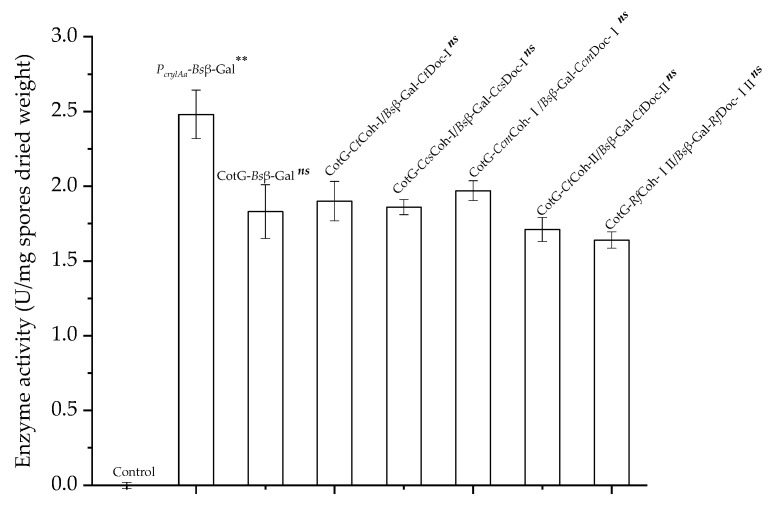
Comparison between the enzyme activities of different spore-displayed *Bs*β–Gals. Control, wild type *B. subtilis* WB600 spores. The values are the means obtained from triplicated experiments. Error bars represent standard deviations. Comparison of data among groups was performed one-way ANOVA with Tukey’s post-hoc test. ** denotes significant difference between *P_cry1Aa_*–*Bs*β–Gal and other six groups (*p* < 0.05); *ns* indicates no significant differences between any two of those groups (CotG–*Bs*β–Gal, CotG–*Ct*Coh-I/*Bs*β–Gal–*Ct*Doc-I, CotG–*Ct*coh-II/*Bs*β–Gal–*Ct*Doc-II, CotG–*Ccs*Coh-I/*Bs*β–Gal–*Ccs*Doc-I, CotG–*Ccm*Coh-I/*Bs*β–Gal–*Ccm*Doc-I and CotG–*Rf*coh-III/*Bs*β–Gal–*Rf*Doc-III) (*p* > 0.05).

To further evaluate the applicability of Coh–Doc based spore display, the efficiency of spore displayed *Bs*β–Gal joined with different Coh–Doc module pairs was compared by examining their activities toward the hydrolysis of *o*-nitrophenyl-β-D-galactopyranoside (ONPG). As shown in Figure 4, all five spores displayed *Bs*β–Gals exhibited activities ranging from 1.64 to 1.97 U/mg spores, with CotG–*Ccm*Coh-I/*Bs*β–Gal–*Ccm*Doc-I (1.97 U/mg spores) being the highest. The activities of *P_cry1Aa_*–*Bs*β–Gal and CotG–*Bs*β–Gal were determined as 2.48 and 1.83 U/mg spores, respectively. Interestingly, wild type *B. subtilis* WB600 spores pre-incubated with *Bs*β–Gal–Doc appears to be able to hydrolyze ONPG compared to those pre-incubated in phosphate buffer (data not shown), indicating that the very small amount of *Bs*β–Gal–Doc adsorbed onto spores was active. Thus, the activity observed with Coh–Doc based spore displayed *Bs*β–Gal is most likely due to the combination of the activities of the spore displayed *Bs*β–Gal and the adsorbed enzyme. Although it was difficult to distinguish between the enzymatic activity due to the spore displayed *Bs*β–Gal or to the adsorbed *Bs*β–Gal, Coh–Doc based spore displayed *Bs*β–Gal demonstrated the potential of Coh–Doc interaction to advance *B. subtilis* spore display platform.

Next, the dot blotting analysis was performed. A comparative study was made between conventional spore display from *P_cry1Aa_*–*Bs*β–Gal or CotG–*Bs*β–Gal and individual Coh–Doc based spore displayed *Bs*β–Gal, respectively CotG–*Ccm*Coh-I/*Bs*β–Gal–*Ccm*Doc-I and CotG–*Rf*Coh-III/*Bs*β–Gal–*Rf*Doc-III. From the results summarized in Table 1, the number of fusion protein molecules per spore was estimated as 1.3×10^5^ for *P_cry1Aa_*–*Bs*β–Gal, almost 3 times higher than that of CotG–*Bs*β–Gal. However, as already noted the activity of *P_cry1Aa_*–*Bs*β–Gal was only about 1.4 times higher than that of CotG–*Bs*β–Gal, the former of which showed a relatively lower display efficiency despite higher expression level, revealing that of the *Bs*β–Gal tethered on the spore surface, a small but quite portion was catalytically inactive.

On the other hand, as Table 1 shows, the number of CotG–*Bs*β–Gal molecules was 4.3 × 10^4^ per spore, which was approximately 1.4-fold higher than that of CotG–*Ccm*Coh-I/*Bs*β–Gal–*Ccm*DocI (3.0 × 10^4^) and 1.5-fold higher than that of CotG–*Rf*Coh-III/*Bs*β–Gal–*Rf*Doc-III (2.8 × 10^4^). Surprisingly, CotG–*Bs*β–Gal showed a relatively low activity than CotG–*Ccm*Coh-I/*Bs*β–Gal–*Ccm*Doc-I, but only 1.1 times higher than that of CotG–*Rf*Coh-III/*Bs*β–Gal–*Rf*Doc-III. Similar to *P_cry1Aa_*–*Bs*β–Gal, CotG–*Bs*β–Gal expression level did not correlate well with its activity. These results implied that Coh–Doc based spore display exhibited a higher display efficiency than the conventional spore display via direct fusion or native protein display.

### 2.5. Enzymatic Properties of Spore Displayed Bsβ–Gal

**Figure 5:**
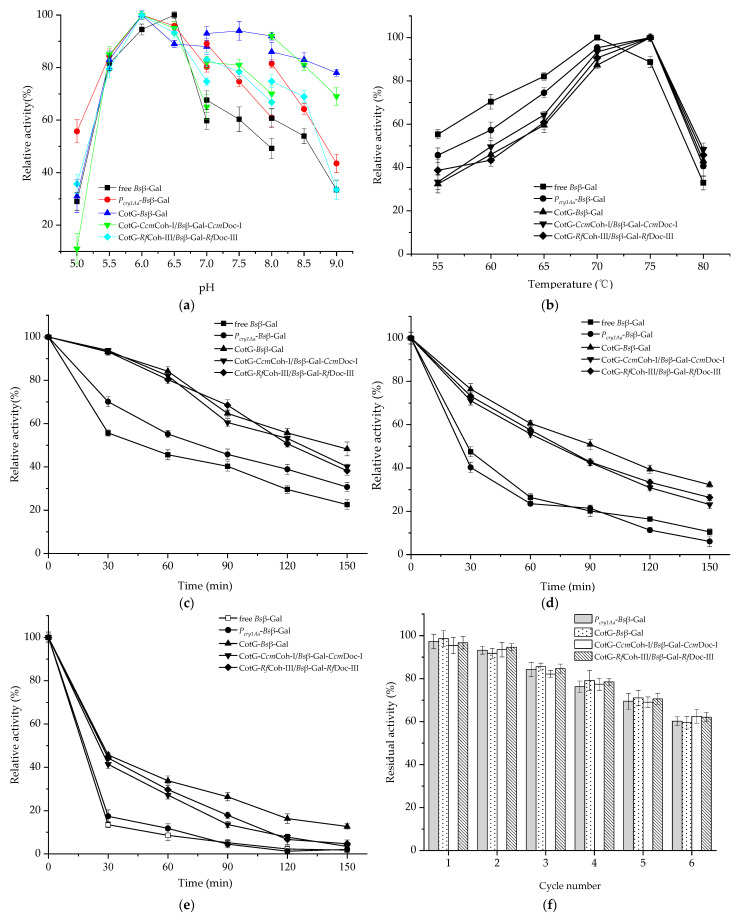
Characterizations of spore-displayed *Bs*β–Gals. (**a**) Optimum pH of spore-displayed and purified *Bs*β–Gals. (**b**) The effect of temperature on the activities of spore-displayed and purified *Bs*β–Gals. Temperature stabilities of spore-displayed and purified *Bs*β–Gals at 60 °C (**c**), 65 °C (**d**) and 70 °C (**e**), respectively. (**f**) Reusability of spore-displayed *Bs*β–Gals. The data are the means of three replicates. Error bars represent standard deviations.

The influences of pH, temperature, and organic solvent on the enzymatic properties were investigated. The purified *Bs*β–Gal displayed the highest activity at pH 6.5. The optimum pH of spore displayed *Bs*β–Gal for ONPG hydrolysis was 6.0 (Figure 5a), which was consistent with the reported results [12]. As Figure 5a shows, CotG–*Bs*β–Gal maintained a relatively high activity level at pH varying from 6.0 to 9.0 as compared with other types of *Bs*β–Gal. *P_cry1Aa_*–*Bs*β–Gal, CotG–*Ccm*Coh-I/*Bs*β–Gal–*Ccm*Doc-I and CotG–*Rf*Coh-III/*Bs*β–Gal–RfDoc-III presented similar pH activity profiles. Notably, free *Bs*β–Gal exhibited a relatively low activity within the pH range of 7.0–9.0, indicating it was unstable at alkaline pH conditions. In short, as for spore displayed *Bs*β–Gal, the optimum pH shifting to an acidic region and high activity obtained in alkaline environment was mainly attributed to the spore resistant features.

The behavior of spores displayed *Bs*β–Gal was also studied in the temperature range of 55 to 80 °C at the optimal pH buffer. The same optimum temperature was found for spore displayed *Bs*β–Gal at 75 °C, which was 5 C higher than that of free *Bs*β–Gal (Figure 5b). Interestingly, free *Bs*β–Gal had a relatively high activity at 55–70 °C, besides broadening. Thermostability of spore displayed *Bs*β–Gal was investigated at 65, 70 and 75 °C for 0–2.5 h. At all the tested temperatures, both free and spore displayed *Bs*β–Gal activities decreased over time (Figure 5c–e). It was found that CotG–*Bs*β–Gal, CotG–*Ccm*Coh-I/*Bs*β–Gal–*Ccm*Doc-I and CotG–*Rf*Coh-III/*Bs*β–Gal–*Rf*Doc-III retained 16.3 ± 2.2%, 7.8 ± 1.1% and 6.7 ± 2.4% residual activities, respectively, after 2 h-incubation at 75 °C, whereas both free *Bs*β–Gal and *P_cry1Aa_*–*Bs*β–Gal nearly lost all of their activities (Figure 5e). Figure 5 also shows that free *Bs*β–Gal and *P_cry1Aa_*–*Bs*β–Gal without anchoring motif displayed similar temperature stability trends, especially at higher temperatures (70 and 75 °C). More importantly, CotG–*Bs*β–Gal, CotG–*Ccm*Coh-I/*Bs*β–Gal–*Ccm*Doc-I and CotG–*Rf*Coh-III/*Bs*β–Gal–*Rf*Doc-III retained higher activities than *P_cry1Aa_*–*Bs*β–Gal and free *Bs*β–Gal. The superior thermostability upon spore display by direct fusion or Coh–Doc affinity could be due to the unique structure and resistance properties of the spore, which allows *Bs*β–Gal to endure high temperature for a longer time.

The activity and stability of spore displayed *Bs*β–Gal was further examined in the presence of organic solvents at a volume ratio of 1:1. As summarized in Table 2, under the specified conditions, the relative activity of free *Bs*β–Gal was positively correlated with the octanol/water partition coefficient of organic solvent (defined as log*P*, which was calculated using online service https://www.molinspiration.com (accessed on 6 January 2021)). This result may be attributed to the higher activity of *Bs*β–Gal in non-polar solvent [13]. Intriguingly, *P_cry1Aa_*–*Bs*β–Gal without anchoring motif has projected a similar organic solvent stability profile as its free *Bs*β–Gal form. Table 2 also shows that spore displayed *Bs*β–Gal through direct fusion or Coh–Doc affinity was able to withstand in most of the tested organic solvents, demonstrating their excellent organic solvent resistance. A similar result was found on the spore displayed β–Gal derived from *E. coli* [13]. Toluene, a representative aromatic organic compound with log*P* of 2.39, maintained 73.3% of the initial activity (CotG–*Bs*β–Gal), 67.4% (CotG–*Ccm*Coh-I/*Bs*β–Gal–*Ccm*Doc-I), and 71.3% (CotG–*Rf*Coh-III/*Bs*β–Gal–*Rf*Doc-III), respectively, whereas only 33.1% of activity was retained for free *Bs*β–Gal. Besides, ethyl ether having log*P* equivalent to 1.05, maintained 56.1% of the initial activity (CotG–*Bs*β–Gal), 47.2% (CotG–*Ccm*Coh-I/*Bs*β–Gal–*Ccm*Doc-I), and 46.5% (CotG–*Rf*Coh-III/*Bs*β–Gal–*Rf*Doc-III), respectively. However, the activity of free *Bs*β–Gal was reduced to 21.4%. All types of *Bs*β–Gals experienced a sharp decline in stability in ethanol and 1,4-dioxane. Moreover, 1,4-dioxane acted as the most detrimental organic solvent to an extent. It was shown that no detectable remaining activity was observed for *P_cry1Aa_*–*Bs*β–Gal and free *Bs*β–Gal. Even for spore displayed forms, only 3.6% (CotG–*Bs*β–Gal), 0.4% (CotG–*Ccm*Coh-I/*Bs*β–Gal–*Ccm*Doc-I), and 1.1% (CotG–*Rf*Coh-III/*Bs*β–Gal–*Rf*Doc-III) of activities were retained.

The recyclable performance of an enzyme is a key issue for practical applications. For assessing the reusability of spore displayed *Bs*β–Gal, a total of six cycles were explored. As shown in Figure 5f, the relative activities of all four types of spore displayed *Bs*β–Gals gradually decreased with increasing recycle numbers, and each of them still remained about 60% of its initial activity after 6 times of recycling. Different types of spore displayed *Bs*β–Gals exhibited similar residual activities after every run. A recent study by Chen et al. [6] described that *E. coli* β–Gal displayed on the spores via Coh–Doc interaction still maintained 80% of its initial activity after four rounds of reuse. Taken together, those observations demonstrated good reusability of the spore displayed *Bs*β–Gal. 

For biocatalysts, calculating the kinetic parameters is crucial for their realistic application. The *K_m_* values of free purified *Bs*β–Gal, *P_cry1Aa_*–*Bs*β–Gal, CotG–*Bs*β–Gal, CotG–*Ccm*Coh-I/*Bs*β–Gal–*Ccm*Doc-I and CotG–*Rf*Coh-III/*Bs*β–Gal–*Rf*Doc-III were measured as 2.97, 5.71, 4.43, 3.83 and 3.71 mM, respectively. The free purified *Bs*β–Gal with a *K_m_* of 2.97 mM for ONPG is consistent with the reported data by Chen et al. [14]. This suggests that spore displayed *Bs*β–Gals with high *K_m_* values are more likely to exhibit a less affinity for artificial substrate ONPG compared with the free enzyme (Table 3). Coh–Doc based spore display, however, exhibited significantly lower *K_m_* than native protein spore display or direct fusion spore display. This phenomenon may be ascribed to the introduction of a specific Coh–Doc pair, which could greatly increase the spatial distance between the target enzyme and the anchoring motif, thereby modulating *Bs*β–Gal’s flexibility necessary for substrate affinity. Moreover, CotG–*Rf*Coh-III/*Bs*β–Gal–*Rf*Doc-III (3.71 mM) and CotG–*Ccm*Coh-I/*Bs*β–Gal–*Ccm*Doc-I (3.83 mM) had the relatively lower *K_m_*, the *V_max_* with the latter (1.04 μmol min^−1^ mg spores^−1^) being approximately 1.3 times that of the former (0.78 μmol min^−1^ mg spores^−1^). In light of enzymes with low kinetic values, the CotG–*Ccm*Coh-I/*Bs*β–Gal–*Ccm*Doc-I could have a potential for practical bioprocess.

## 3. Discussion

Demand in the global enzyme market has greatly increased as a result of the growing population and exhaustion of natural resources. The utilization of enzymes is rendered more favorable in industrial processes especially by enzyme immobilization. In recent decades, great advances in the field of enzyme immobilization have made immobilized enzymes very promising catalysts in modern industry. However, enzyme immobilization (especially chemical immobilization) is still facing many challenges if analyzed in a global way, such as the overall cost of the supports, the ease of the immobilization protocols and protein conformation sensitivity [15,16]. Developing novel immobilization approaches to improve enzyme performance has been a permanent pursuit. Surface display of enzymes on the *B. subtilis* spores presents an alternative for enzyme immobilization.

Though *B. subtilis* spore display offers several advantages over the existing enzyme immobilization methods, the efficiency of display systems is very low. This restricts the application of *B. subtilis* spore display as biocatalysts. Thus, constructing an efficient *B. subtilis* spore display platform for enzyme immobilization is necessary from both the theoretical and applied sciences point of view. To date, however, few reports are aimed at improving display efficiency. Recently, displaying target protein on the spores via high-affinity Coh–Doc interaction has been demonstrated to be a promising approach. Chen et al. [7] only examined the possibility of type I Coh-Doc complexes from *C. thermocellum* and *R. flavefaciens* to immobilize enzymes on the spores. Other two Coh-Doc interactions, namely type II and type III, were not tested. The universality of Coh-Doc interaction for the spore display of biologically active molecules should be further confirmed by more studies. The efficiency of different types of *B. subtilis* spore display was previously recorded in separate studies on β-galactosidases from *B. stearothermophilus* IAM11001 and *E. coli* [7,12,17]. The aim of this study is thus to gain more insights into the efficiency of Coh–Doc based spore display in comparison with the conventional spore display via direct fusion or native protein display. A key for developing such spore display may be the screening of suitable Coh–Doc modules. In a previous report, a type I Coh–Doc pair originating from *C. thermocellum* ATCC 27405 was applied to construct *E. coli* β–Gal spore display system, wherein the fusion gene cotG–coh was directly integrated into the *B. subtilis* genome [7]. To achieve improved performance, herein, three kinds of Coh–Doc pairs (namely type I, type II, and type III) were chosen, and an episomal plasmid pEB03 was employed. To minimize the steric hindrance and endow the enzyme with more flexibility, a suitable linker was fused between *Bs*β–Gal and its dockerin module. In doing so, *Bs*β–Gal was successfully immobilized on the surface of recombinant *B. subtilis* WB600 spores through CotG as an anchoring motif via Coh–Doc interaction.

The effect of type of Coh–Doc on display efficiency was investigated. CotG–*Ccm*Coh-I/*Bs*β–Gal–*Ccm*Doc-I exhibited significant improvement in display efficiency compared to those of conventional spore display, suggesting the critical role of incorporation of a suitable Coh–Doc module in enhancing display efficiency. This result could be ascribed to the following aspects: (1) three types of Coh–Doc interactions exhibited different distribution, specificity and structure; (2) the binding of cohesin from mesophilic *C. cellulovorans* DSM 743B to counterpart dockerin is highly selective, whereas in *C. thermocellum* ATCC 27405 and *C. cellulolyticum* ATCC 35319 the cohesin interacted at random with available cohesin of scaffoldins [18,19]; (3) cellulosome produced by *C. thermocellum* ATCC 27405 was observed to be larger than those for *C. cellulovorans* DSM 743B and *C. cellulolyticum* ATCC 35319 [20]; (4) a type III Coh–Doc interaction was only discovered in *R. flavefaciens* FD-1 [21].

Generally, the dockerin fused *Bs*β–Gal that is tethered to the spore surface through Coh-Doc interaction has high flexibility to react with substrate. Moreover, a better distribution of *Bs*β–Gal–Doc on the spore surface may give rise to more efficient interaction with substrate. Therefore, by introducing Coh–Doc module pair between CotG and *Bs*β–Gal, which could weaken the spatial confinement effect and alter the distribution of CotG-fused protein, the display efficiency of CotG–*Bs*β–Gal was effectively improved. A previous study has documented that overproduced CotG protein tended to accumulate at the midpoint of the spores. Hypothetically, a considerable amount of CotG–*Bs*β–Gal may embed at the midpoints of the spores, being difficultly accessed to substrate molecules and hampering its catalytic activity. Similar to enzyme immobilization, the relatively lower display efficiency is sometimes associated with changes in the CotG–*Bs*β–Gal enzyme structure [22].

Despite the high expression level, the decreased display efficiency of *P_cry1Aa_*–*Bs*β–Gal over those of the Coh–Doc based spore display or CotG–*Bs*β–Gal would imply that the former had a lower proportion of active enzyme on the spore surface. Furthermore, *P_cry1Aa_*–*Bs*β–Gal was not effectively protected from high temperatures and organic solvents. It has been hypothesized that the proteins in an embedded state could reduce the display efficiency of enzymes but afford considerable protection to displayed enzymes. In most instances, the inactivation of multimeric enzymes is dissociation of the enzyme subunits or loss of their correct assembly structure [23]. Based on these observations, a possible explanation might be that *P_cry1Aa_*–*Bs*β–Gal was overproduced during the display process, which resulted in an undesired fold of some enzyme molecules into an inactive form or the dissociation of enzyme subunits.

The thermostability assay revealed that spore-displayed *Bs*β–Gal via direct fusion or Coh–Doc interaction possessed enhanced thermostability compared to its native form. Importantly, CotG–*Bs*β–Gal could attain a relatively higher degree of protection than CotG–*Ccm*Coh-I/*Bs*β–Gal–*Ccm*Doc-I. By displaying on the spores, a significant enhancement in thermostability was also found on the β–Gal of *E. coli* that fused with CotG [13]. Other enzymes, such as organophosphorus hydrolase and haloalkane dehalogenase, when displayed as a fusion with CotG, were reported to exhibit higher thermostability than their free forms [24,25]. The ability to withstand organic solvent is another sought after feature, as it would benefit real bioprocess. Our data showed that both direct fusion and Coh-Doc based spore displayed *Bs*β–Gal could be more tolerable to organic solvent than *P_cry1Aa_*–*Bs*β–Gal and the free enzyme. Similar to thermostability, there is no discernible difference in organic solvent resistance between *P_cry1Aa_*–*Bs*β–Gal and free *Bs*β–Gal. In general, the spore surface of *B. subtilis* is highly hydrophobic [26], and hydrophobic and electrostatic interactions are the main driving forces of spore passive adsorption [27]. The spore displayed *Bs*β–Gal with no anchoring motif (*P_cry1Aa_*–*Bs*β–Gal) was unstable when exposed to organic solvents. This instability could in part be elucidated by the hydrophobic effect of organic solvents resulted by the detachment of some *Bs*β–Gal adsorbed on the spore surface. Besides, changes in environment (such as temperature and organic solvent) may destabilize the hydrophobic and electrostatic interactions between *Bs*β–Gal and spore surface structure and cause the dissociation of subunits, leading to its inactivation.

From these results, the extreme robustness as well as the thermal and organic solvent stability of spore displayed *Bs*β–Gal is due to the spore inert structure, and the interaction between the displayed enzyme and the proteins in the coat layer. In particularly, the latter may strengthen the stability of *Bs*β–Gal multimeric structure and prevent subunit dissociation, thereby resulting in better catalytic performance. Furthermore, analysis of the effects of temperature and organic solvent on the stability of *P_cry1Aa_*–*Bs*β–Gal had depicted that native protein display without anchoring motif was unable to exhibit superior performance over direct fusion spore display or Coh–Doc based spore display thereby limiting its potential use in practical applications.

In this paper, we further investigated the Coh–Doc interaction-based display method that could effectively improve the display performance of *Bs*β–Gal on the spores by evaluating the efficiency and technical feasibility of three kinds of Coh–Doc interactions. This is the first time that such an elaborate comparison has been made between conventional spore display and several of their individual Coh–Doc-based spore displays from four microbes. In particularly, the observation that the same *Bs*β-gal was displayed on the spores with different efficiencies by Coh-Doc of different origins or different types of the same origin, suggests that these Coh-Doc modules have crucial role in constructing *B. subtilis* spore display. In another word, the most appropriate Coh-Doc have to be identified for each enzyme to be immobilized and for each specific application. This, on the other hand, reflects the importance of our work to scientific field of *B. subtilis* spore display. As indicated in this work, Coh–Doc from *C. cellulolyticum* ATCC 35319 exhibited the highest display efficiency and substrate affinity; thus, it can be considered as a preferable choice for enzyme immobilization.

## 4. Materials and Methods

### 4.1. Bacterial Strains and Growth Conditions

All bacterial strains and plasmids used are listed in Table S1. *E. coli* (DH5α or BL21(DE3)), used for gene cloning and protein production, were cultured in LB broth. *B. subtilis* WB600 was used as display host. Sporulation was triggered by nutrient deprivation in Difco sporulation medium (DSM). Spore preparation was performed as described before [12]. Media were supplemented with ampicillin, kanamycin, or spectinomycin when necessary.

### 4.2. Construction of Spore Display Plasmid and Its Transformation

A list of primers used in the experiments is shown in Table S2. The genes encoding type I and type II cohesins (*Ct*coh-I and *Ct*coh-II) of *C. thermocellum* ATCC 27405 were amplified from its genomic DNA by PCR, followed by separate insertion into a previously constructed non-integrative plasmid pEB03-cotG to create pEB03-cotG-*Ct*coh-I and pEB03-cotG-*Ct*coh-II, respectively. Similarly, a type I cohesin from *C. cellulovorans* DSM 743B (*Ccs*coh-I), a type I cohesin from *C. cellulolyticum* ATCC 35319 (*Ccm*coh-I) and a type III cohesin from *R. flavefaciens* FD-1 (*Rf*coh-III) were PCR amplified and cloned into pEB03-cotG to generate pEB03-cotG-*Ccs*coh-I, pEB03-cotG-*Ccm*coh-I and pEB03-cotG-*Rf*coh-III, respectively. For the construction of a direct fusion-based spore display system (CotG–*Bs*β–Gal), full-length *bgaB* encoding *Bs*β–Gal was cloned by amplifying it from pJS-cotY-bgaB [28]. pEB03-cotG-bgaB was constructed similarly to that described for pEB03-cotG-*Ct*coh-I. To develop a native protein spore display system (*P_cry1Aa_*–*Bs*β–Gal), a sporulation-specific promoter *P_cry1Aa_* was chemically synthesized based on the previous results [17], and inserted into an earlier constructed plasmid pEB03-bgaB [12], resulting in the pEB03-P_cry1Aa_-bgaB. Each construct was fused with a FLAG tag at the C-terminus for detection purpose. After verifying by restriction digestion and DNA sequencing, all of the plasmid constructs were transformed into competent wild type *B. subtilis* WB600 cells. Transformants were screened on LB plate containing 300 μg/mL spectinomycin and further checked by colony PCR analysis.

### 4.3. Expression and Purification of Bsβ–Gal–Doc

Cloning of *bgaB* was performed by PCR from pJS-cotY-bgaB followed by ligation into pET-28a, yielding pET-28a-bgaB. Two kinds of dockerin genes, namely a type I dockerin and a type II dockerin, were first PCR amplified from *C. thermocellum* ATCC 27405 and cloned into pET-28a-bgaB to give pET-28a-bgaB-*Ct*doc-I and pET-28a-bgaB-*Ct*doc-II, respectively. Similarly, a type I dockerin gene of *C. cellulovorans* (*Ccs*doc-I), a type I dockerin gene of *C. cellulolyticum* (*Ccm*doc-I) and a type III dockerin gene of *R. flavefaciens* (*Rf*doc-III) were PCR amplified and inserted into pET-28a-bgaB to generate pET28b-bgaB-*Ccs*doc-I, pET28b-bgaB-*Ccm*doc-I and pET28b-bgaB-*Rf*doc-III, respectively. The colony PCR and DNA sequencing were used to confirm the insertion and the identity of the recombinant clones. Finally, the correct recombinant plasmids were transformed into *E. coli* BL21(DE3). 

*Bs*β–Gal expression was induced by 0.6 mM IPTG at 30 °C for 10 h. The harvested cells were collected by centrifugation, resuspended in 20 mM Tris-HCl buffer and then disrupted by ultrasonication with 20 min pulses at 20 kHz. After heat treatment at 80 °C for 20 min, the crude enzyme was purified through a Ni-NTA Prepacked chromatographic column (Sangon Biotech, Shanghai, China). The detailed procedure for separation of the enzyme extract was followed the product instruction’s guide. The active fractions were pooled and dialyzed against 20 mM Tris-HCl. The purify of *Bs*β–Gal was detected by SDS-PAGE.

### 4.4. Bsβ–Gal Assembly on the Spore Surface

To confirm the assembly of *Bs*β–Gal on the spore surface, the *Bs*β–Gal was fused with five different dockerin modules and expressed in *E. coli*. After cells were cultured in DSM at 37 °C for 48 h, the *B. subtilis* spores expressing a fusion of CotG and cohesin were harvested using the described method before [28]. Each of the obtained recombinant spores displaying CotG–*Ct*Coh-I, CotG–*Ct*Coh-II, CotG–*Ccs*Coh-I, CotG–*Ccm*Coh-I and CotG–*Rf*Coh–III (1.0 × 10^8^ CFU/mL) were incubated with the corresponding purified dockerin-fused *Bs*β–Gal (0.05 mg/mL), i.e., *Bs*β–Gal–*Ct*Doc-I, *Bs*β–Gal–*Ct*Doc-II, *Bs*β–Gal–*Ccs*Doc-I, *Bs*β–Gal–*Ccm*Doc-I and *Bs*β–Gal–*Rf*Doc-III, respectively. The binding assay was conducted at 37 °C for 30 min under gentle shaking. At last, the nonspecifically bound proteins were removed by washing the spores thrice with phosphate-buffered saline (PBS, pH 7.0).

### 4.5. Western Blotting and Dot Blotting Analyses

For western blotting analysis, the coat proteins were extracted by treatment of the purified recombinant spores with 1% (*w/v*) SDS and 50 mM dithiothreitol as described elsewhere (Cutting and Van der Horn [29]). Western blotting analysis was done as follows. The first step was to confirm the surface expression of CotG–Coh fusion protein on the spores using rabbit anti–FLAG antibody as a primary antibody. The extracted spore coat proteins of different recombinant strains were separately subjected to SDS-PAGE, followed by western blotting with horseradish peroxidase (HRP)–conjugated goat anti-rabbit IgG. The blots were developed with HRP–DAB substrate kit to visualize protein band. Next, to validate the immobilization of *Bs*β–Gal-Doc on the spores via interaction with CotG–Coh, extracted spore coat proteins of different recombinant strains were separated on 12% SDS-PAGE, and then analyzed on a western blotting, in which signals were developed with rabbit polyclonal anti–*Bs*β–Gal antibody. Dot blotting experiment was applied to further quantify the amount of CotG–Coh/*Bs*β–Gal-Doc in the spore coat following the procedures of Wang et al. [12].

### 4.6. Immunofluorescence Microscopy 

Procedures of the immunofluorescence experiment were virtually the same as formerly described [28]. The sample was probed using rabbit anti–FLAG antibody or rabbit anti–*Bs*β–Gal antibody, followed by Alexa Fluor 488-conjugated goat anti-rabbit IgG secondary antibody. Images were captured using a SP2 confocal microscope system (Leica, Mannheim, Germany).

### 4.7. Assay of Spore Displayed Bsβ–Gal Activity 

The activity of dockerin-fused *Bs*β–Gal was measured using ONPG as a specific substrate. The reaction mixture contained 800 μL of 2.5 g/L ONPG in 20 mM potassium phosphate buffer (pH 6.0) and 200 μL of prepared spore suspension. After 5 min of incubation at 75 °C, 1 mL of 10% (*w*/*v*) sodium carbonate was added to stop the reaction. The amount of the produced *o*-nitrophenol (ONP) was determined by measuring absorbance at 420 nm. The wild type *B. subtilis* WB600 spores were used as a negative control. One unit of spore-anchored *Bs*β–Gal activity was described as the amount of 1 μM ONP per minute under the specified conditions.

### 4.8. Characterization of Spore Displayed Bsβ–Gal

The impact of pH on the activity of spore displayed *Bs*β–Gal was investigated by using phosphate citrate buffer (pH 5.0–7.0), potassium phosphate buffer (pH 7.0–8.0), and Tris-HCl buffer (pH 8.0–9.0) at a concentration of 0.1 M. The optimum temperature was determined at temperatures of 55, 60, 65, 70, 75 and 80 °C. Thermostability was evaluated according to the protocol described by Wang et al. [12]. The effect of organic solvents (ethyl ether, toluene, 1-chloroheptane, *n*-hexane, acetonitrile, ethanol, cyclohexanone and 1,4-dioxane) on the stability of spore displayed *Bs*β–Gal was examined by subjecting the enzyme along with organic solvents to pre-incubation at 25 °C for 1 h. Besides, the reuse stability and determination of kinetic constants of different CotG-based *Bs*β–Gal spore display systems were also characterized. The kinetic parameters of spore displayed *Bs*β–Gals were investigated by using varying concentrations of ONPG (0.83–20 mM) as substrate. The kinetic data were fitted to the Lineweaver-Burk equation and the Michaelis constant (*K_m_*) and maximum velocity (*V_max_*) values were obtained.

## Figures and Tables

**Table 1:** Quantification of *Bs*β–Gal expression by dot-blot analysis.

*Bs*β–Gal Source	Amount of Sample Used	Density in OD/mm^2^ (± SD)	β–Gal Concentration (ng)	Number of Fusion Protein Molecules per Spore
Free *Bs*β–Gal	10.0 ng	50.3 (± 0.12)	NA	
	5.0 ng	25.8 (± 0.04)	NA	
	2.5 ng	12.9 (± 0.06)	NA	
CotG–*Ccm*Coh-I/*Bs*β–Gal–*Ccm*Doc-I	2.0 × 10^6^	42.2 (± 0.07)	8.4	3.0 × 10^4^
	1.0 × 10^6^	22.1 (± 0.02)	4.4	
	5.0 × 10^5^	10.9 (± 0.11)	2.2	
CotG–*Rf*Coh-III/*Bs*β–Gal–*Rf*Doc-III	2.0 × 10^6^	39.8 (± 0.13)	7.9	2.8 × 10^4^
	1.0 × 10^6^	19.6 (± 0.12)	3.9	
	5.0 × 10^5^	11.4 (± 0.08)	2.3	
CotG–*Bs*β–Gal	1.0 × 10^6^	36.6 (± 0.06)	7.3	4.3 × 10^4^
	5.0 × 10^5^	19.1 (± 0.11)	3.8	
	2.5 × 10^5^	9.8 (± 0.04)	2.0	
*P_cry1Aa_*–*Bs*β–Gal	5.0 × 10^5^	42.3 (± 0.06)	8.4	1.3 × 10^5^
	2.5 × 10^5^	21.1 (± 0.09)	4.2	
	1.3 × 10^5^	11.9 (± 0.05)	2.4	

**Table 2:** Stabilities of spore displayed and free *Bs*β–Gals in various organic solvents.

Solvent	log*P* Value of Solvent	Relative Activity (%)				
		Free *Bs*β-Gal	*P_cry1Aa_*–*Bs*β–Gal	CotG–*Bs*β–Gal	CotG–*Ccm*Coh-I/*Bs*β–Gal–*Ccm*Doc-I	CotG–*Rf*Coh-III/*Bs*β–Gal–*Rf*Doc-III
Control		100 ± 0.8	100 ± 1.3	100 ± 1.8	100 ± 0.9	100 ± 1.6
*n*-Hexane	3.66	93.4 ± 2.1	93.7 ± 2.7	102.4 ± 3.4	98.3 ± 3.5	97.2 ± 2.4
1-Chloroheptane	3.37	90.6 ± 2.5	91.8 ± 2.9	107.8 ± 1.7	99.3 ± 2.2	98.5 ± 3.1
Toluene	2.39	33.1 ± 1.1	37.5 ± 1.6	73.3 ± 2.5	67.4 ± 1.4	71.2 ± 1.8
Cyclohexanone	1.40	37.2 ± 1.3	39.3 ± 1.9	67.8 ± 2.2	62.9 ± 2.7	66.1 ± 2.0
Ethyl ether	1.05	21.4 ± 0.7	27.9 ± 1.4	56.1 ± 2.6	47.2 ± 1.3	46.5 ± 1.5
Acetonitrile	0.47	12.3 ± 0.4	11.4 ± 0.2	30.6 ± 0.5	28.2 ± 0.6	29.5 ± 1.0
Ethanol	0.06	0	1.7 ± 0.1	13.2 ± 0.3	6.5 ± 0.4	8.3 ± 0.2
1,4-Dioxane	−0.23	0	0	3.6 ± 0.1	0.4 ± 0.1	1.1 ± 0.1

The log*P* of various organic solvents was predicted using the toolkit at the site: www.molinspiration.com (accessed on 6 January 2021). The results were calculated with three replicates and expressed as mean ± standard deviation (SD).

**Table 3:** The kinetics constants of four different types of spore displayed *Bs*β–Gals.

Recombinant Spores	*V*max/(μmol·min^−1^·mg·spores^−1^ (dry weight))	*K*m/(mM)
*Bs*β–Gal	7.24 ± 0.12 ^a^	2.97 ± 0.05 ^a^
*P_cry1Aa_*–*Bs*β–Gal	1.01 ± 0.16 ^b^	5.71 ± 0.09 ^b^
CotG–*Bs*β–Gal	0.77 ± 0.13 ^b^	4.43 ± 0.16 ^c^
CotG–*Ccm*Coh-I/*Bs*β–Gal–*Ccm*Doc-I	1.04 ± 0.08 ^b^	3.83 ± 0.10 ^d^
CotG–*Rf*Coh-III/*Bs*β–Gal–*Rf*Doc-III	0.78 ± 0.11 ^b^	3.71 ± 0.17 ^d^

The results were calculated with triplicated tests and expressed as mean ± standard deviation (SD). Means within the same column with different superscript letters are significantly different (*p* < 0.05).

## Data Availability

The data presented in this study are available within the article.

## Supplementary Materials

The following are available online, Table S1: Strains and plasmids used in this study, Table S2: List of all primers used in the study.
