# Supplementary material for: Constructing an Efficient Bacillus subtilis Spore Display by Using Cohesin−Dockerin Interactions
Source: Molecules. 2021 Feb 23;26(4):1186. doi: 10.3390/molecules26041186 (PMC7926950; doi:10.3390/molecules26041186)
Supplement: Supplementary file 1 [file molecules-26-01186-s001.pdf]

Supplementary materials for

Journal name: Molecules

Manuscript Title: Constructing an efficient *Bacillus subtilis* spore display by using cohesin−dockerin interaction

He Wang [1,*,†], Xiaomin Jiang [2,†], Yongchang Qian [3], and Lianghong Yin [3]

[1] School of Grain Science and Technology, Jiangsu University of Science and Technology, Zhenjiang 212100, Jiangsu, China

[2] School of Agricultural and Food Sciences, Zhejiang Agriculture and Forestry University, Hangzhou 311300, Zhejiang, China; 821767674@qq.com

[3] School of Forestry and Biotechnology, Zhejiang Agriculture and Forestry University, Hangzhou 311300, Zhejiang, China; qian3906@zafu.edu.cn (Y.Q); ylh4@163.com (L.Y)

* Correspondence: wh2989@just.edu.cn; Tel.: +86-511-84423559

† These authors contributed equally to this work.

Table S1. Strains and plasmids used in this study

Name	Description	Source
Strains		
E. coli		
Top10	F^{-} *mcrA Δ(mrr-hsdRMS-mcrBC) φ80lacZΔM15 ΔlacX74 nupG recA1 araD139 Δ(ara-leu)7697 galE15 galK16 rpsL*(Str^{R}) *endA1* λ^{-}	Laboratory stock
BL21(DE3)	F^{-} *ompT gal dcm lon hsdSB* ($r_B^{-}m_B^{-}$) *λ(DE3 [lacI lacUV5-T7 gene 1 ind1 sam7 nin5])*	Laboratory stock
Clostridium thermocellum ATCC 27405	Template for encoding type-I and type-II cohesin genes	ATCC
Clostridium cellulovorans DSM 743B	Template for encoding type-I cohesin gene	DSMZ
Clostridium cellulolyticum ATCC 35319	Template for encoding type-I cohesin gene	ATCC
Ruminococcus flavefaciens FD-1	Template for encoding type-III cohesin gene	DSMZ
B. subtilis		
168	*trpC2*	BGSC
WB600	*ΔnprE, ΔnprB, aprΔ3, Δepr, Δbpf, ΔaprE, trpC2*	BGSC
GC01	WB600WT *cotG-Ctcoh-I*	This study

GC02	WB600WT *cotG-Ctcoh-II*	This study
GC03	WB600WT *cotG-Ccscoh-I*	This study
GC04	WB600WT *cotG-Ccmcoh-I*	This study
GC05	WB600WT *cotG-Rfcoh-III*	This study
BD01	BL21(DE3) *bgaB-Ctdoc-I*	This study
BD02	BL21(DE3) *bgaB-Ctdoc-II*	This study
BD03	BL21(DE3) *bgaB-Ccsdoc-I*	This study
BD04	BL21(DE3) *bgaB-Ccmdoc-I*	This study
BD05	BL21(DE3) *bgaB-Rfdoc-III*	This study
GBJ01	WB600WT *cotG-Ctcoh-I*::*bgaB-Ctdoc-I*	This study
GBJ02	WB600WT *cotG-Ctcoh-II*::*bgaB-Ctdoc-II*	This study
GBJ03	WB600WT *cotG-Ccscoh-I*::*bgaB-Ccsdoc-I*	This study
GBJ04	WB600WT *cotG-Ccmcoh-I*::*bgaB-Ccmdoc-I*	This study
GBJ05	WB600WT *cotG-Rfcoh-III*::*bgaB-Rfdoc-III*	This study
GB01	WB600WT *cotG-bgaB*	This study
PB01	WB600WT P_{cry1Aa}-*bgaB*	This study

Plasmids		
pET-28a	pBR322 *ori lacI T7lac Kan*^{r}	Novagen
pET-28a-*bgaB*	pET-28a carrying *bgaB*	(Wang et al., 2016)
pET-28a-*bgaB-Ctdoc-I*	pET-28a carrying fusion gene *bgaB-Ctdoc-I*	This study
pET-28a-*bgaB-Ctdoc-II*	pET-28a carrying fusion gene *bgaB-Ctdoc-II*	This study
pET-28a-*bgaB-Ccsdoc-I*	pET-28a carrying fusion gene *bgaB-Ccsdoc-I*	This study
pET-28a-*bgaB-Ccmdoc-I*	pET-28a carrying fusion gene *bgaB-Ccmdoc-I*	This study
pET-28a-*bgaB-Rfdoc-III*	pET-28a carrying fusion gene *bgaB-Rfdoc-III*	This study
	E. coli-B. subtilis shuttle plasmid	(Xu et al., 2011)
pEB03-*cotG*	pEB03 carrying *cotG*	(Wang et al., 2016)
pEB03-*cotG-Ctcoh-I*	pEB03 carrying fusion gene *cotG-Ctcoh-I*	This study
pEB03-*cotG-Ctcoh-II*	pEB03 carrying fusion gene *cotG-Ctcoh-II*	This study
pEB03-*cotG-Ccscoh-I*	pEB03 carrying fusion gene *cotG-Ccscoh-I*	This study
pEB03-*cotG-Ccmcoh-I*	pEB03carrying fusion gene *cotG-Ccmcoh-I*	This study
pEB03-*cotG-Rfcoh-III*	pEB03 carrying fusion gene *cotG-Rfcoh-III*	This study

pEB03-*cotG-bgaB*	pEB03 carrying fusion gene *cotG-bgaB*	This study
pEB03-P_{cry1Aa}-*bgaB*	pEB03 carrying P_{cry1Aa}-*bgaB*	This study

Table S2. List of all primers used in the study

Name	Sequence (5′→3′)	Restriction enzyme site
cotG-F	ATCGATAAGCTTGATATCGAATTCCTTTTCTAGAAGTGTCCCT	*EcoR* Ⅰ
cotG-R	AGAACTAGTGGATCCCTGCAGTTTGTATTTCTTTTTGACTACCCAGCAATTG	*Pst* Ⅰ
coh Ⅰ-1-*F*	AAAAAGAAATACAAACTGCAGGGAGATACAACAGTACCTAC	*Pst* Ⅰ
coh Ⅰ-1-*R*	CGCTCTAGAACTAGTGGATCCTTACTTGTCGTCATCGTCTTTGT	*Bam*H Ⅰ
coh Ⅱ-1-*F*	AAAAAGAAATACAAACTGCAGATTGAAATGGTTCTTGATAA	*Pst* Ⅰ
coh Ⅱ-1-*R*	CGCTCTAGAACTAGTGGATCCTTACTTGTCGTCATCGTCTT	*Bam*H Ⅰ
coh Ⅰ-2-*F*	AAAAAGAAATACAAACTGCAGGTAACAGCTACAATTGGAAAAGT	*Pst* Ⅰ
coh Ⅰ-2-*R*	CGCTCTAGAACTAGTGGATCCTTACTTGTCGTCATCGTCTTTGTAGTCGATAGTTACTGTTCCTGGGT	*Bam*H Ⅰ
coh Ⅰ-3-*F*	AAAAAGAAATACAAACTGCAGGAAATATCAATCGGCAAAGT	*Pst* Ⅰ
coh Ⅰ-3-*R*	CGCTCTAGAACTAGTGGATCCTTACTTGTCGTCATCGTCTTTGTAGTCTGTTATCTCACCCTCTGTGA	*Bam*H Ⅰ
*coh*Ⅲ-1-*F*	AAAAAGAAATACAAACTGCAGGGAGATACAACAGTACCTAC	*Pst* Ⅰ
*coh*Ⅲ-1-*R*	CGCTCTAGAACTAGTGGATCCTTACTTGTCGTCATCGTCTTTGT	*Bam*H Ⅰ
1-*bgaB-F*	AAAAAGAAATACAAACTGCAGATGAATGTGTTATCCTCAATTTGTTACGG	*Pst* Ⅰ
1-*bgaB-R*	CGCTCTAGAACTAGTGGATCCTTACTTGTCGTCATCGTCTTTGTAGTCAACCTTCCCGGCTTCATCATGC	*Bam*H Ⅰ
2-*bgaB-F*	GTGCCGCGCGGCAGCCATATGATGAATGTGTTATCCTCAATTTGTTACGG	*Nde* Ⅰ
2-*bgaB-R*	ATCGCCCCATGTAGGGAATTCACTACCGCCACCTCCAACCTTCCCGGCTTCATCATGC	*Eco*R Ⅰ
doc Ⅰ-1-*F*	GGAGGTGGCGGTAGTGAATTCCCGAAAATTACCTATGGAGA	*Eco*R Ⅰ
doc Ⅰ-1-*R*	GTGGTGGTGGTGGTGCTCGAGAACGGGAAAACTCGTTATTA	*Xho* Ⅰ

*doc*Ⅱ-1-*F*	GGAGGTGGCGGTAGTGAATTCATAATGATGTGGGTAGGAGA	*Eco*RⅠ
*doc*Ⅱ-1-*R*	GTGGTGGTGGTGGTGCTCGAGTGCGTCGTAATCACTTGATG	*Xho*Ⅰ
*doc*Ⅰ-2-*F*	GGAGGTGGCGGTAGTGAATTCATAATGATGTGGGTAGGAGA	*Eco*RⅠ
*doc*Ⅰ-2-*R*	GTGGTGGTGGTGGTGCTCGAGGCTAAGAAGTTTCTTTTTTAGAAGAG	*Xho*Ⅰ
*doc*Ⅰ-3-*F*	GGAGGTGGCGGTAGTGAATTCGTAATTGTATATGGAGATTA	*Eco*RⅠ
*doc*Ⅰ-3-*R*	GTGGTGGTGGTGGTGCTCGAGGCTTGGAAGCTTACTTACCA	*Xho*Ⅰ
*doc*Ⅲ-1-*F*	GGAGGTGGCGGTAGTGAATTCCCTACATGGGGCGATACAAA	*Eco*RⅠ
*doc*Ⅲ-1-*R*	GTGGTGGTGGTGGTGCTCGAGCTAAACGTCTGCGTTAACCTTAC	*Xho*Ⅰ
